# Prevention norms across socio-ecological levels and their relationship to PrEP-intention among men who have sex with men: a structural equation model

**DOI:** 10.1186/s12889-026-28207-1

**Published:** 2026-07-22

**Authors:** Estelle Thunnissen, Jeroen De Man, Veerle Buffel, Edwin Wouters

**Affiliations:** 1 Centre for Population, Family and Health, University of Antwerp, Antwerp, Belgium; 2 University of the Western Cape, Cape Town, South Africa; 3 Brussels Institute for Social and Population Studies, Vrije Universiteit Brussel, Brussels, Belgium; 4 Centre for Health Systems Research & Development, University of the Free State, Bloemfontein, South Africa

**Keywords:** HIV, PrEP, MSM, Social norms, Socio-ecological model

## Abstract

**Background:**

Social norms around PrEP act as both barriers to and stimulants of the use of PrEP across a wide range of target populations, yet remain relatively understudied. To stimulate theoretical innovation and provide a quantitative check for qualitative findings, we integrate norm types from the Behavioral Models (BM) and the Social Norms Approach (SNA) into the socio-ecological model (SEM) and analyze their relationship with PrEP-intention. We include attitude and role belief at the individual level, injunctive norms at the interpersonal level and descriptive norms and self-comparison to peers at the community level.

**Methods:**

The data were drawn from a survey on sexual health distributed on social media by Flemish MSM community organizations from October 19^th,^ 2022, to December 19^th,^ 2022, resulting in a total convenience sample of 610. Structural Equation Modelling analyses, including confirmatory factor analysis of the latent variables and path analysis of the relationship between norm types and PrEP intention, were conducted on a convenience sample of 350 Belgian MSM who were not living with HIV.

**Results:**

At the individual level, attitude towards PrEP was positively and moderately to strongly associated with intention, while role belief that ‘It is inappropriate for someone with my relationship(s) to use PrEP’ was negatively and moderately to strongly associated with intention. At the interpersonal level, injunctive norms, the expectation of a positive reaction to the respondent’s PrEP use, were positively and moderately to strongly associated with intention. At the community level, descriptive norms, thinking more peers use PrEP, were positively and weakly associated with intention. While self-comparison to peers, estimating one’s own sexual behaviour as safer than peers, was negatively and weakly associated with intention.

**Conclusion:**

All norm types included in the analysis, at the individual level (attitude and role belief), interpersonal level (injunctive norms), and the community level (descriptive norms and self-comparison to peers), were associated with PrEP-intention among MSM. Most PrEP interventions target cognition, while our analysis shows significant untapped potential for norm-based interventions to increase intention to use PrEP.

**Supplementary Information:**

The online version contains supplementary material available at https://doi.org/10.1186/s12889-026-28207-1.

## Introduction

Continued strengthening of HIV prevention is critical, as more than 1.5 million people acquire HIV annually [1]. Pre-Exposure Prophylaxis (PrEP) has the potential to be a game changer because of its high clinical efficacy and the feasibility of uptake among key populations such as Men who have Sex with Men (MSM) [2]. Yet, despite its potential, uptake remains suboptimal. Globally, a 2023 meta-analysis estimated that only 16% of PrEP-eligible MSM had ever used PrEP, with incremental increases over time (Huang, 2023). In Belgium, where this study is set, an estimated 18% of eligible MSMused PrEP in 2018 [3]. Understanding the factors that facilitate or hinder PrEP uptake is thus crucial to closing this gap and maximizing population-level prevention. To this end, studies have often used proxies such as acceptability, willingness to use, and intention to use PrEP. Importantly, acceptability or willingness mainly captures attitudes, which are only one component of intention and subsequent behavior [4]. While evidence on the intention–behavior relationship is mixed, meta-analyses indicate that intention is among the strongest predictors of behavior [5, 6], and this is also the case for PrEP use [7].

Comprehensive qualitative research highlights that social norms play a pivotal role in shaping PrEP-related behaviors. They can act as both barriers and facilitators to willingness, intention, uptake, and adherence across diverse populations, including MSM, transgender women, cisgender women at high risk, and people who inject drugs [8–14]. Social norms –defined as “a predominant behavioral pattern within a group, supported by a shared understanding of acceptable actions and sustained through social interactions within that group.” [15] provide a framework that guides individual prevention practices by establishing what is collectively considered acceptable and expected within a community.

A lot of quantitative research in this domain focuses on individual sexual behavior and socio-demographic characteristics as correlates of PrEP uptake [11, 16–25]. However, recently, a series of studies has used a behavior model (BM) framework to explain PrEP behavior from a more comprehensive socio-psychological perspective [24, 26–40]. Such HIV prevention analyses can draw on more than 30 Behavioral Models (BM), and a wide range of social norm constructs is available across these models [41, 42]. So far, BM analyses of PrEP have been limited to taking a general Social Cognitive Theory approach, or variations of the Theory of Planned Behavior, Health Belief Model, Theory of Information-, Motivation- Behavioral skills model, Self-Determination Theory, and Identity Process Theory [27–37]. These behavioral model studies largely focus on cognitive components or individual-level norms, such as attitudes, while a series of very recent studies extend the framework to account for broader community and social influences [43, 44] or to situate behavioral determinants within multi-level socio-ecological contexts [29, 44–47].

Broader prevention research and qualitative PrEP studies suggest that all overlapping socio-ecological levels must be considered when examining barriers to preventive behavior [43, 48–50]. Bronfenbrenner’s socio-ecological model (SEM) highlights the interplay between individual, relational, community, and societal factors, emphasizing that effective prevention requires coordinated action across multiple levels [51]. Norms are central across these levels, shaping both individual behavior and community participation [7, 9, 10]. Studying PrEP-related norms and their interactions across ecological levels is therefore essential to inform interventions that promote sustained uptake and establish collective acceptance of PrEP as an effective prevention strategy. Consequently, in this study, we aim to provide an answer to the following research question (RQ): “How do social norms at the individual, interpersonal, and community level relate to intention to use PrEP among MSM in Belgium?” We aim to provide a more nuanced understanding of how social norms influence prevention behavior by integrating norms distinguished in Behavioral Models (BM) and the Social Norms Approach (SNA) into the SEM. Recent articles reviewing the field of behavioral models (BM) call for such theoretical integration to revitalize health behavioral research [41, 52]. SNA starts from the hypothesis that the perception of our peers’ behavior and opinions can drive us to align our own actions with what we believe our peers typically do and think, and has mostly been applied to alcohol use [53], with select studies considering these mechanisms in light of PrEP [43, 54–58]. SNA’s strength lies in its focus on norms at the interpersonal (injunctive norms) and community levels (descriptive norms). Injunctive norms refer to our perception of what others think, while descriptive norms refer to our perception of what others do. These norms are also part of the Information–Motivation–Behavioral Skills Model and are important for predicting PrEP willingness, intention, and use [57, 59]. SNA’s limitation is that it includes only two norm types and lacks a type at the individual level. BMs, on the other hand, often include norms at the individual level and have a theoretical basis for a broader range of norm types than SNA, but their focus typically remains on cognitive factors [41, 42, 52]. We integrate three non-overlapping norm-types from BM that make important contributions to the prediction of prevention-related behavioral intentions, namely: attitude, role belief, and self-comparison to peers, also called reference group and peer models [60–63].

At the individual level of the SEM, we include attitude and role belief from BM [26, 27, 29, 30, 33–36, 54, 64, 65]. Attitude refers to a relatively enduring organization of beliefs, feelings, and behavioral tendencies towards socially significant objects [29]. Previous studies found attitude towards PrEP positively associated with willingness, information seeking, intention, and use [26, 27, 29, 30, 33–36, 64, 65].

- Hypothesis 1: A person’s attitude towards PrEP is positively associated with intention to use PrEP.

Role belief refers to individuals’ perceptions of the accepted behavior for people who hold certain positions in society, and which role is pertinent is extremely context-dependent [60]. A belief that condoms are inappropriate for a role an individual is fulfilling was found to be negatively associated with their condom use [60] and qualitative evidence suggests the same mechanism may be true for PrEP [61, 66]. Role-identity can affect behavior that is stronger than an individual’s attitudes or perceived social norms [67]. For example, Wulfert et al. (1996) found that the belief that condom use was inappropriate for one’s age impacted condom use among gay men in the US in the nineties [62], while Dillon at (2024) found that masculine gender role beliefs of machismo impacted PrEP use among Latin American MSM in 2024 [44].

- Hypothesis 2: A belief that condoms are inappropriate for a role an individual is fulfilling is negatively associated with intention to use PrEP.

At the interpersonal level, we include injunctive norms from the SNA. Injunctive norms refer to the perceived approval of others for a given behavior. A majority of studies on PrEP also found injunctive norms positively associated with willingness, information seeking, intention, and uptake, while a minority found no effect [27, 30, 33, 35, 36, 64, 65, 68, 69].

- Hypothesis 3: Injunctive norms are positively associated with intention to use PrEP.

At the community level, we include descriptive norms from the SNA and self-comparison to peers from BM. Descriptive norms refer to perceptions of others’ engagement in behaviors [70]. Previous studies on PrEP uniformly found descriptive norms positively associated with either willingness, information seeking, intention, and use [26, 29, 34, 36, 37, 65, 68, 69].

- Hypothesis 4: Descriptive norms are positively related to intention to use PrEP.

Self-comparison to peers refers to the mechanism that comparing one’s own behavior to that of peer models can result in imitation of the behavior observed [62, 63]. A perception that one has safer sex than others was found to be negatively associated with condom use, and we explore if the same mechanism holds for PrEP. Qualitative research has demonstrated that self-comparison to others significantly impacts perceptions of and uptake of PrEP [71].

- Hypothesis 5: Self-comparison to peers is negatively associated with intention to use PrEP.

**Fig. 1:**
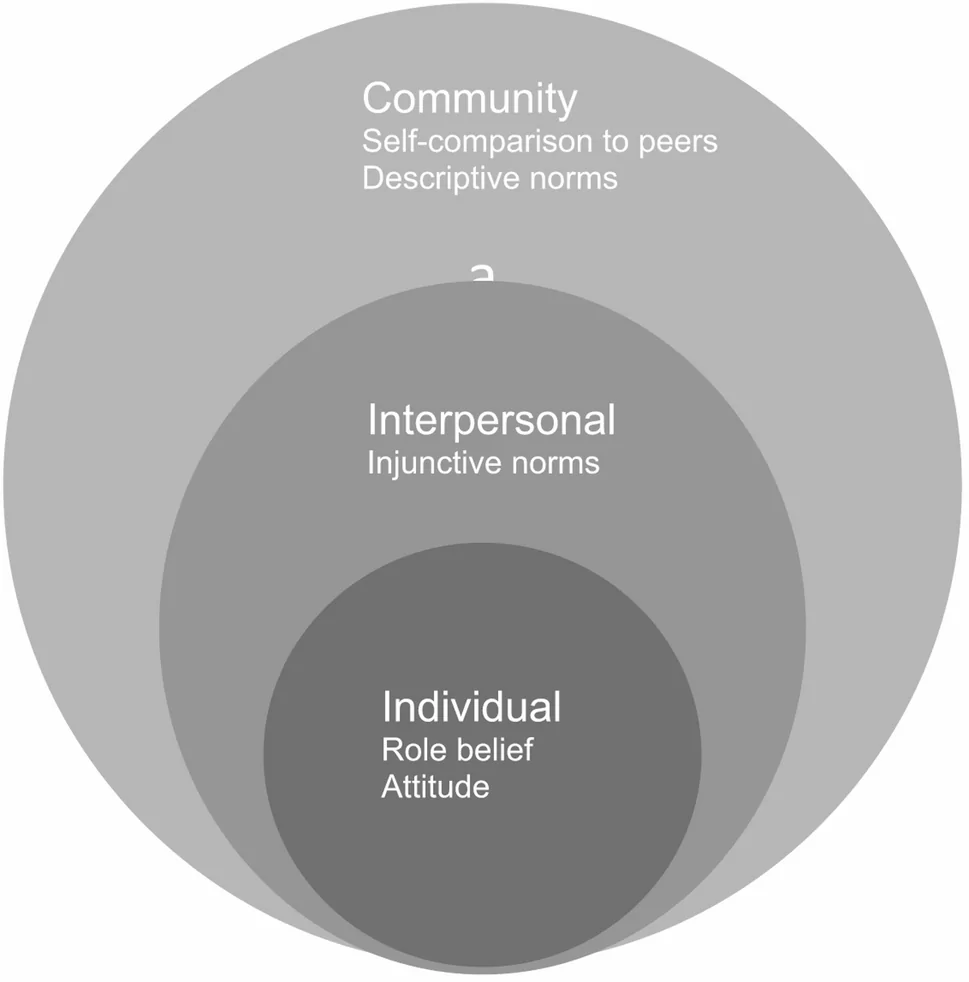
Norm-types nested within the socio-ecological model

For an overview of our full conceptual model, see Fig. 1. For an overview of our hypotheses, see Table 1.

**Table 1:** Hypotheses

Hypothesis 1	A person’s attitude towards PrEP is positively associated with intention to use PrEP.
Hypothesis 2	A belief that condoms are inappropriate for a role an individual is fulfilling is negatively associated with intention to use PrEP.
Hypothesis 3	Injunctive norms are positively associated with intention to use PrEP.
Hypothesis 4	Descriptive norms are positively associated with intention to use PrEP.
Hypothesis 5	Self-comparison to peers is positively associated with intention to use PrEP.

## Methods

### Research context

In Belgium, PrEP is delivered through 12 HIV reference centers (HRC). HRCs are specialized outpatient clinics providing multidisciplinary HIV and PrEP care. PrEP is partially reimbursed for individuals with greater exposure to HIV, identified through the national eligibility criteria [72, 73].

### Participants

The data were drawn from a Dutch, French, and English-language survey on sexual health distributed on social media by Flemish MSM community organizations from October 19th, 2022, to December 19^th,^ 2022, resulting in a total convenience sample of 610. Exclusion criteria were being cis-female, not having sex with men, being under 18 years of age, and not currently living in Belgium. Participation was entirely voluntary, and no financial incentives were offered, minimizing any motivation for falsification. For this paper, we take a subsample of 350 MSM who indicated they were not living with HIV or were unaware of their status, and who were not currently using PrEP. Institutional ethical review and approval were obtained from the University of Antwerp (SHW_20_47, December 2021). We obtained consent from all participants.

### Measures

Questions were developed through a multi-step process involving research team members, PrEP experts, and community stakeholders from the MSM community in Flanders and the Brussels-Capital Region. Given the extreme research fatigue of our target population [74] and the need to adapt existing questions to the Belgian MSM context, we actively involved community stakeholders in developing the questionnaire. They emphasized the importance of keeping the questionnaire short and thus shortening scales and measuring some concepts with single questions. The process was as follows: (1) initial development of a preliminary list of potential questions based on BM and SNA theory and norm concepts measured in previous prevention studies [26–30, 33–37, 61, 64, 65, 68, 69] as well as qualitative findings on PrEP norms [71, 75–78]; (2) review of the initial list in focus groups with social scientists with PrEP expertise and representatives of MSM community organizations; (3) adaptation and refinement of the questions based on feedback from the focus groups. (4) pilot testing of the questionnaire among MSM community organization representatives and further modifications based on their feedback; (5) pilot testing among members of the target population; and (6) final modifications of the questions based on feedback from the pilot with the target population.

PrEP intention: Willingness and intention are often conflated in research on prevention intention, and a way to mitigate this is to ask about specific behavior of increasing difficulty within a specified time span [4]. Participants were asked how likely they were to look for more information about PrEP in the next 12 months, to talk to a healthcare worker about PrEP in the next 12 months, and to start using PrEP in the next 12 months. Answers were on a Likert scale from 1 (“very unlikely”) to 5 (“very likely”). Response items were combined as constructs.

Attitude: Participants were asked how much they agreed with six statements measuring components of an individual’s attitude toward PrEP, with responses on a Likert scale from 1 (“strongly disagree”) to 5 (“strongly agree”). The items were as follows: “People who take PrEP sleep around”, “I would not trust someone who told me he is on PrEP”, “I would disapprove that a friend takes PrEP”, “If I was looking for a sex partner I would prefer to have sex with someone who takes PrEP”, “People who take PrEP are responsible” and “PrEP users have made a smart decision to protect their health”.

Role belief: Role belief is highly context-dependent, and through our iterative process, we found that the role of a partner (sexual/steady/romantic/friendly/trusted) is most relevant to PrEP use among MSM in Belgium, with particular emphasis on the role of a steady partner. Role belief in this study refers to a belief that a person’s relationship type determines the appropriateness of PrEP use. It was measured using one item; participants were asked how much they agreed with the following statement: “It is inappropriate for someone with my relationship(s) to use PrEP”. Response options ranged from 1 = ‘strongly disagree’ to 5 = ‘strongly agree’. To explore whether people in steady relationships feel that taking PrEP is especially inappropriate for that type of partnership, we include the relationship between having one or more steady partners and role belief in our model. We asked participants to indicate all that currently applies: “I have sex with…”. With answer categories: “one steady partner”, “multiple steady partners”, “one fuck buddy”, multiple fuckbuddies”, “occasional partners”, and “I don’t have sex”. Our community stakeholders indicated that ‘fuck buddies’ were partners one has sex with regularly and who one is friends with, while ‘occasional partners’ referred to people one only has sex with infrequently (including only once) and with whom they do not maintain a friendship.

Injunctive norms: Participants were asked, “How would your family react if you told them you take PrEP?”, “How would your closest gay friends or acquaintances react if you told them you take PrEP?”, and “How do you think gay men your age would react if you told them you take PrEP?”. Response options ranged from 1 = “very negatively” to 5 = “very positively”.

Descriptive norms were measured using one question: “How many gay men your age do you think take PrEP?” with answers (1) “almost no one”, (2) “a small part”, (3) “about half”, (4) “a large part”, (5) “almost all”.

Self-comparison to peers: Participants were asked to rate how safe (protection against HIV and STIs) the sex was that they have and that gay men their age have, on a scale of 1 (very unsafe) to 10 (very safe). The final construct score, self-comparison to peers, was computed by subtracting the peer safety score from the personal safety score.

Based on theory and previous research, we selected a set of covariates [79]. How these were measured was based on the EMIS questionnaire [80], adapted to fit the context of the study. The model includes dummy variables for education (professional education or higher), age (younger than 35), degree of urbanity (> 10.000 residents), and greater exposure to HIV [79]. Greater exposure to HIV risk was operationalized as having more than one sex partner and engaging in condomless anal sex.

### Statistical analyses

Structural equation modeling analyses were conducted in R using the packages “lavaan” and “semTools” [81, 82]. The package “semTools” was used to obtain specific reliability measures and measures of fit not offered by the lavaan package [82]. First, a confirmatory factor analysis was conducted for each latent construct to evaluate whether it is adequately measured by its hypothesized Likert-scale items. To this end, we assessed the fit of the measurement model for each latent variable and the loadings of the item indicators on the latent variables. To assess model fit, the comparative fit index (CFI), the Tucker-Lewis index (TLI), and the root mean squared error of approximation (RMSEA) were examined. CFI and TLI values above 0. 95, RMSEA values below 0.06, and a standardized root mean square residual (SRMR) below or equal to 0.08 represent a good fit. Second, we conducted a path analysis using a structural equation modeling framework that integrated the measurement models from the confirmatory factor analyses. Model specification was based on theory and informed by data-driven modification indices. The same model fit indices were used to assess the final model’s fit. Potential confounders were identified based on theoretical assumptions, with model complexity and overfitting considered. They were simultaneously added as covariates to the final model, and changes in model fit were examined to ensure the model remained adequate. All analyses employed diagonally weighted least squares to estimate the model parameters, combined with the full weight matrix to compute robust standard errors, and a mean- and variance-adjusted test statistic [4].

**Fig. 2:**
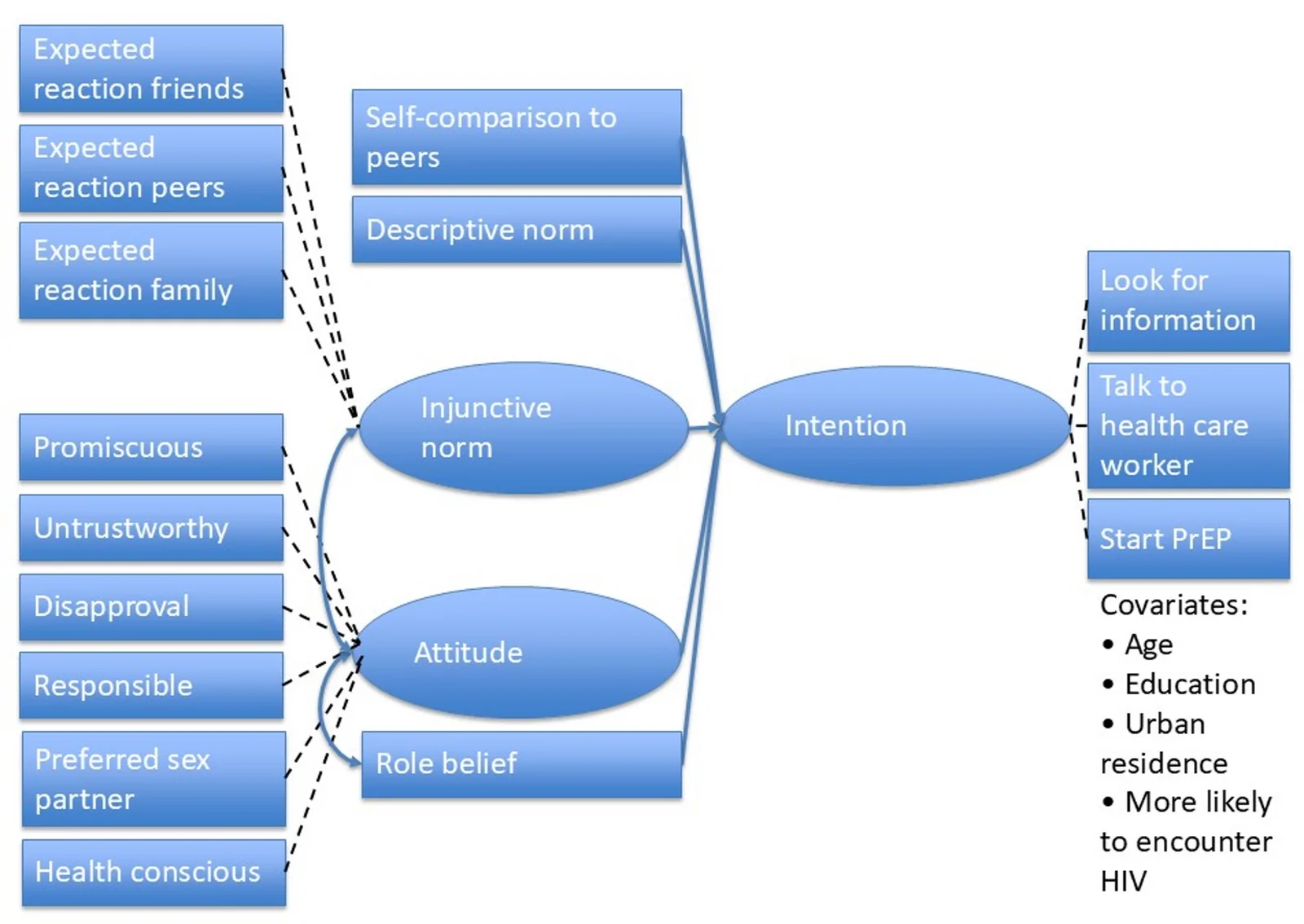
Structural equation model

In the model, attitude and role belief, as well as attitude and injunctive norms, were allowed to covary. We prioritized covariates that were conceptually most relevant and supported by the literature [62]. This approach ensured that the model captured the key sources of variation while maintaining parsimony. For a visual representation of the model, see Fig. 2. To assess model goodness of fit, the comparative fit index (CFI), the Tucker-Lewis index (TLI), and the root mean squared error of approximation (RMSEA) were examined. CFI and TLI values above 0. 95, RMSEA values below 0.06, and a standardized root mean square residual (SRMR) below or equal to 0.08 represent a good fit.

Multivariate imputation by chained equations with predictive mean matching was used to handle missing data under the missing-at-random assumption. For most variables, including the outcome variables (i.e., intention to use PrEP), the percentage of missing values was below 5%. The variables related to injunctive norms, descriptive norms, self-comparison to peers, steady partner(s), and greater exposure to HIV had between 10 and 13% missing. Rubin’s rules were used to pool points and SE estimates across 12 imputed data sets. The procedure was done using the “Mice” package in R [83].

## Results

### Participant socio-demographic characteristics

Of the 350 respondents included in the final sample, all reported being a gay or bisexual man, transwomen or non-binary. Nearly all, 334 (95%), identified as male. A majority, 305 (87%), reported only having sex with men. The median age was 40 years (SD = 12 years), and more than half (205, 59%) were over 35 years of age. Half of the participants, 179 (51%), graduated from a technical college or university. Regarding place of residence, 185 (53%) lived in a rural area or small town with fewer than 10.000 inhabitants. About half (188, 54%) reported being in one or more steady relationships. About one third, 114 (33%), were more likely to encounter HIV.

### Measurement model

Our model comprised three latent factors: intention, attitude, and injunctive norms. The item ‘People who take PrEP sleep around’ was excluded from the final Attitude scale, supported by four considerations: (1) the item demonstrated a weak factor loading (λ = -0.45), (2) modification indices indicated problematic error covariances with other items (MI ≤ 18), (3) SRMR reduced from 0.063 to 0.056 upon the item’s exclusion, (4) The item’s ambiguous and subjective wording raised concerns about content validity. Factor loadings (λ) were all significant (*P* < 0.001). To view the complete list of items, loadings, and reliability indices, see Table 2. The model fit indices for the measurement models met the criteria for good fit.

**Table 2:** Measurement model loadings and reliabilities

	Standardized Factor Loading	z-value
Intention to use PrEP (AVE*= 0.818)		
How likely is it that you will do the following:		
-Look for more information about PrEP in the next 12 months	0.86	**
-talk to a healthcare worker about PrEP in the next 12 months	0.96	37.80
-start using PrEP in the next 12 months	0.93	44.01
Attitude (AVE*=0.534)		
How much do you agree with the following statements:		
-I would not trust someone who told me he is on PrEP	-0.68	-15.85
-I would disapprove that a friend takes PrEP	-0.78	-17.34
-If I was looking for a sex partner I would prefer to have sex with someone who takes PrEP	0.48	10.23
-People who take PrEP are responsible	0.82	**
-PrEP users have made a smart decision to protect their health	0.80	17.63
Injunctive norms (AVE*=0.578)		
-How would your family react if you told them you take PrEP	0.35	**
-How would your closest gay friends or acquaintances react if you told them you take PrEP	0.87	5.76
-How do you think gay men your age would react if you told them you take PrEP	0.76	6.08

* Avariage Variance Extracted **items constrained for identification purposes

### Structural equation modeling

**Fig. 3:**
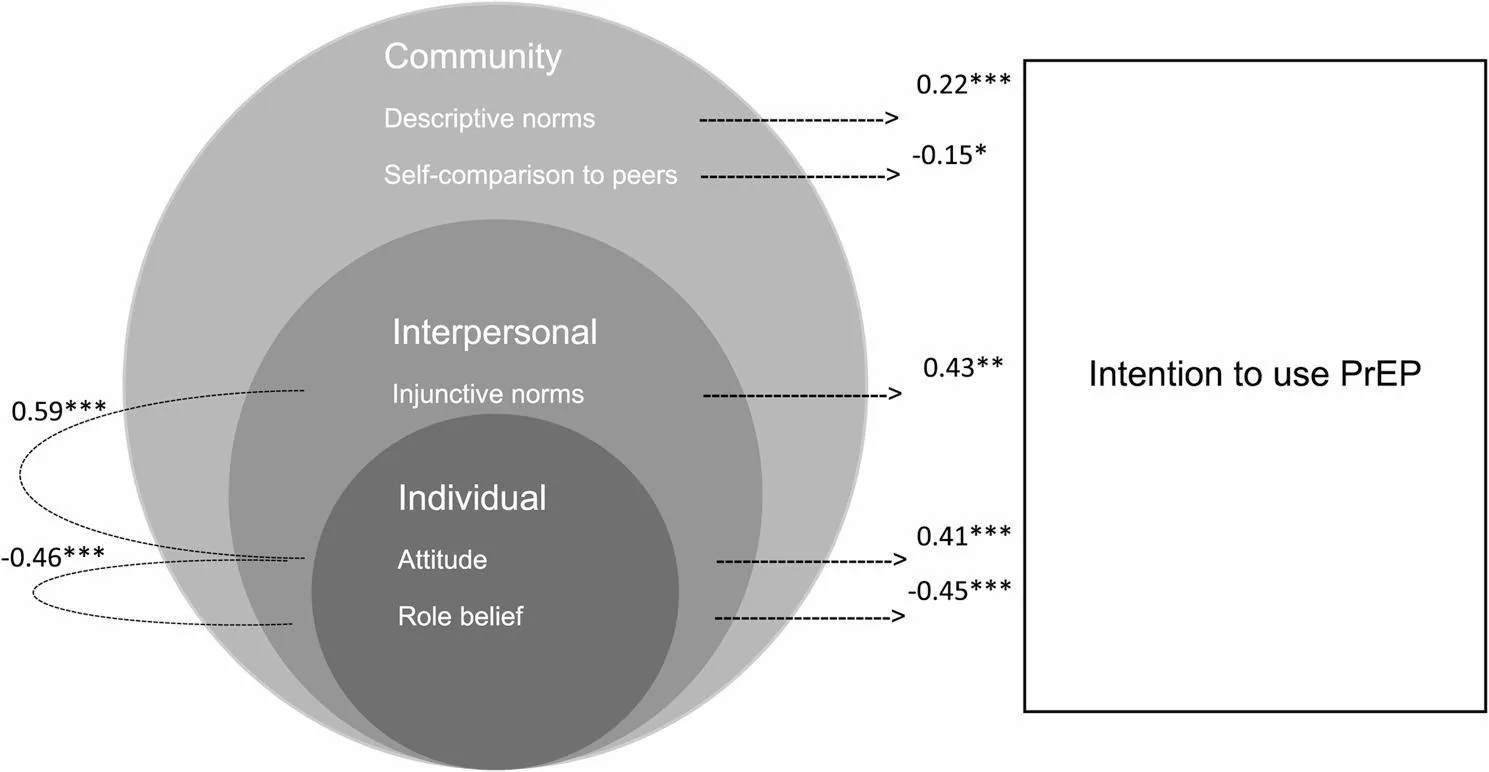
Intention to use PrEP; covariances and total effects. *= *p*<0.05, **=*p*<0.01***=*p*<0.001

Our model converged with acceptable fit: Chi-square = 291, df = 125, RMSEA = 0.067 (90% CI: 0.057–0.076, probability of RMSEA < = 0.005 = 0.004); CFI = 0.968; TLI = 0.983; SRMR = 0.074. See Fig. 3; Table 3 for the exact standardized total effect estimates (coefficients), t-values, and significance level of the hypothesized relationship.

**Table 3:** Structural equation modeling coefficients, t-values and significance level

Hypothesized Relationships	Standardized Total effect Estimates (t-values)
Individual level	
Attitude ◊ Intention	0.41*** (4.93)
Role belief ◊ Intention	-0.45*** (-8.34)
Interpersonal level	
Injunctive norms ◊ Intention	0.43**(2.61)
Community level	
Descriptive norms ◊ Intention	0.22 *** (3.88)
Self-comparison to peers ◊ Intention	-0.15* (-2.52)
Relationships between norms	Covariances
Attitude ⇓◊ Role belief	-0.46*** (-6.85)
Attitude ⇓◊ Injunctive norms	0.59*** (4.66)

*=*p* < 0.05, **=*p* < 0.01***=*p* < 0.001

Of the covariates, only being more likely to encounter HIV was significantly related to intention to use PrEP (Estimate = 0.389; p-value < 0.001). Education, urban living environment and age were not significantly related to intention to use PrEP. For more information on the covariates, see Appendix I.

At the individual level, a person’s attitude towards PrEP had a moderate to strong positive association with intention to use PrEP, with a Standardized Total effect Estimate (β) of 0.41(p-value < 0.001), confirming hypothesis 1. While role belief that ‘It is inappropriate for someone with my relationship(s) to use PrEP’, had a moderate to strong negative association with intention to use PrEP (β=-0.45; p-value < 0.001), confirming hypothesis 2. Being in one or more steady relationships was weakly but significantly associated with a stronger endorsement of the role belief that PrEP use is inappropriate for someone in one’s relationship situation (covariance = 0.19, *p* < 0.01; estimate not shown in tables or figures). Attitude and Role belief also covaried, with a correlation of -0.46 (p-value < 0.001, see Fig. 3).

At the interpersonal level, injunctive norms were moderately to strongly positively related to intention to use PrEP (β = 0.43, p-value < 0.01), confirming hypothesis 3. Injunctive norms also covaried with attitude (covariance = 0.5, p-value < 0.001). Expecting a more positive reaction from family, friends, and peers to one’s own PrEP use is related to a more positive attitude towards PrEP.

At the community level, descriptive norms were weakly positively associated with intention to use PrEP (β = 0.22, p-value < 0.001), confirming hypothesis 4. It is worth noting that, because of the question formulation, descriptive norms were age-specific, with a median age of 40 years (SD = 12 years) and more than half (59%) over 35 years of age. Self-comparison to peers, estimating one’s own sexual behavior as safer than peers, was weakly and negatively associated with intention to use PrEP (β=-0.15, p-value < 0.05), confirming hypothesis 5.

## Discussion

We have found that norms at the individual, interpersonal, and community levels directly impact intention to use PrEP, and that norms across levels are related. Most BM-based PrEP interventions target cognition in one form or another, either by increasing PrEP knowledge, providing tools for risk assessment, creating a decisional balance between the advantages and disadvantages of PrEP, or increasing self-efficacy [7]. Our analysis of the influence of norms at the individual, interpersonal, and community levels on PrEP intentions showed that there is significant untapped potential for norm-based interventions. We recommend the co-created development and testing of interventions targeting norms over multiple socio-ecological levels to create consensus about PrEP as an accepted prevention strategy among MSM.

At the individual level, we found a positive relationship between attitude and intention, which confirms earlier studies where attitude was significantly associated with willingness, information seeking, intention and uptake [26, 27, 29, 30, 35, 36, 64, 65, 69]. Adding nuance, we also found that role beliefs at the individual level and injunctive norms at the interpersonal level covary with attitude, suggesting new paths to boosting positive attitudes toward PrEP. At the interpersonal level, we found a significant, positive, and relatively large relationship between injunctive norms and PrEP-intention. At the community level, we found that descriptive norms have a significant, relatively large effect on intention and self-comparison to peers weakly and negatively associated with intention to use PrEP.

The nuance provided by our analyses can inform intervention design. The question is, how can we better understand and address how MSM feel about PrEP and their perceptions of current prevention norms? One avenue is co-creating PrEP social norms messaging campaigns to increase PrEP uptake. In their review of common pitfalls of social norms approach interventions Cislaghi and Heise (2018) describe the dangers of designing SNA interventions from the outside in. They recommend that practitioners design people-led interventions that help people develop internal motivations to change local norms and strategies to do so in ways compatible with the cultural and social context [84]. Non-norm-based co-created messaging campaigns already exist to promote PrEP adherence and uptake, and PEP uptake [85–88]. With one study on PrEP adherence co-creating m-health messages [86], and another using a crowd-sourcing open call and further co-creation to generate social media-based intervention materials [87]. The intervention on PEP uptake used interviews and focus groups with MSM to develop bus shelter ads and low- to no-cost social media ads [85]. While the campaign aimed at improving PrEP uptake was developed through a co-design process, involving behavioral scientists, clinicians, graphic designers and MSM [88].

Co-created interventions could leverage the potential of combining SNA messaging with messaging grounded in behavioral norms. Campaigns based on the SNA approach spread correct information about the opinions (injunctive norms) and behavior (descriptive norms) of others to promote a consensus on healthy behavior, and studies have shown this can have positive effects on population preventive behavior [70]. While BM studies have shown the impact of attitude, role belief, and self-comparison to peers on intention to engage in preventative behaviors [62].

Most current interventions take place at the individual level, and several have attitude as an outcome [7]. As we found that role belief at the individual level, and injunctive norms at the interpersonal level covary with attitude, interventions might provide information about the number of MSM who feel positively about PrEP and about PrEP use in steady relationships to boost attitude towards PrEP. However, this recommendation comes with a caveat, described below.

At the interpersonal level, current interventions are either peer-model-based or social network-based [7], but they generally do not explicitly target norms. We found that injunctive norms impacted PrEP intention at this level. The majority of studies found injunctive norms significantly related to willingness, information seeking, intention, and uptake [26, 30, 33, 35, 36, 65]. Targeting injunctive norms, however, would only be beneficial if there is a misperception of others’ negative attitudes towards PrEP. If the majority actually feels negatively about PrEP, practitioners may first need to change the attitudes of a core group and help them become change agents, reaching out to the larger group [84]. As for how to target injunctive norms, an intervention aimed at increasing condom use found that an enhanced counseling program positively impacted injunctive norms [89]. Correcting misperceptions of others’ attitudes can also be achieved, for example, by using a web-based application to test social misunderstandings and receive accurate information, by distributing posters with accurate information, and by presenting accurate information in counselling groups [90]. Information on descriptive norms, the behavior of others, can be presented in a similar manner, and in practice, injunctive norms and descriptive norms are often addressed within the same intervention [90]. It is worth noting that one study tested normative messaging promoting PrEP-related information and found that presenting injunctive norms can appear intrusive and backfire [59]. Another study found that injunctive norms only begin to play a role when an individual is actively analyzing information to make preventive decisions [68, 91]. Future studies should investigate the potentially intrusive nature of injunctive norm messaging and the role of injunctive norms during the active PrEP-decision-making process.

Norm-based interventions at the community level aimed at PrEP uptake are rare [7, 88] even though we found descriptive norms at that level have a significant and relatively large effect on intention. While currently marketing and awareness campaigns are common, they lack norm-based messaging [92]. Our results corroborate earlier findings, where descriptive norms were universally significantly and positively associated with willingness, information seeking, intention, and uptake of PrEP [29, 33, 34, 36, 37, 65, 68, 69]. A study piloting a co-created advertisement campaign found that ads that used descriptive norms in the form of statistics about the popularity of PrEP were clicked on more often than other developed adds, and were evaluated more positively [88]. Another study tested normative messages that promote PrEP-related information seeking, manipulated descriptive norms, and manipulated the type of pronoun (individual “you” vs. collective “we”). People favored the use of descriptive normative appeal and collective pronouns in the design of normative messages. For health campaigns targeting MSM’s PrEP-related behaviors, this suggests that information on how many MSM use PrEP and the use of the ‘we’ pronoun may increase PrEP behavior [59, 93].

Participants’ perceptions of their own sexual behavior relative to that of their peers (self-comparison) appeared to influence their PrEP intentions. Consistent with prior work [94, 95], most respondents—around three-quarters—perceived themselves as engaging in safer behavior than their peers, a belief that is unlikely to reflect reality barring extreme sample bias. Drawing on the SNA and Social Cognitive Theory, Wulfert (1996) showed that perceiving others’ sexual behavior as riskier than one’s own can reduce motivation to adopt preventive measures, such as condom use [62]. Our finding extend this mechanism to PrEP: individuals who viewed themselves as safer than their peers reported lower intention to use PrEP. Importantly, this association remained after accounting for HIV exposure, highlighting that the effect is not simply a reflection of actual risk. While qualitative research suggested this comparative mechanism [71], our study is the first to quantitatively demonstrate it for PrEP. These results show that HIV risk perception is not solely a cognitive process but is shaped by social comparisons, situating individual intentions within a broader social context. This has practical implications: interventions that provide accurate information about others’ sexual risk and preventive behaviors may improve personal risk assessment and, in turn, enhance PrEP uptake. Traditional interventions typically focus on individual risk and self-assessment; our findings suggest that supplementing these approaches with peer-contextualized information could increase their effectiveness [7].

Currently, multilevel interventions are extremely rare. Our results show that norms at the individual, interpersonal, and community levels all affect PrEP intention, and that these norms do not function separately; rather, their effects are intertwined and complementary. Instead of interventions aimed at either individuals, networks, or the community, an intervention that targets two or three levels is likely to have a higher impact. SNA typically targets both interpersonal (injunctive) and community (descriptive) norms, but this may be combined with targeting attitudes and role beliefs at the individual level. Wang et al. (2022) found only 2 interventions across multiple socioecological levels in their systematic review of PrEP interventions in the US [7]. These interventions showed that addressing PrEP determinants across multiple socioecological levels is an effective approach to promote PrEP use among MSM [96] but did not target norms. The intervention included in-person group sessions, community-level events, a mobile health app, and a dedicated service provider. Norm-based messaging may be incorporated into such a design.

The aim of our study was to explore PrEP norms across socio-ecological levels and to provide an essential step toward more confirmatory studies; however, our design has several limitations. First, the relatively small sample did not allow us to include correlations among all norm-types in the SEM, so we did not fully explore these relationships. Second, our sample was a convenience sample collected online via a link distributed by MSM community organizations, which almost certainly introduced selection bias. Third, using a cross-sectional design, we found an association between PrEP norms and PrEP intention, but we could not examine whether changes in norms over time are associated with changes in intention to take PrEP, and vice versa. Future longitudinal studies can build on our preliminary findings to further examine this important sexual health mechanism. Fourth, considering that the median age was 40 (SD = 12) and over 50% of participants were above 35, the results may be age-specific. It may be that younger MSM hold different values concerning PrEP, have different perceptions of the values of others, and have a different repertoire of prevention techniques. For example, a study on PrEP perspectives among participants 29–63 years of age found 3 distinct age cohorts: those who began their sexual life before 1981 (pre-HIV era), between 1981 and 1995 (HIV era, pre-ARV), and after 1995 (post-ARV era) [97]. Intention varies significantly between age groups, emphasizing distinct behaviors and perceptions that may influence the decision to use PrEP [98]. As MSM age, changes in sexual practices, social networks, and risk perception often occur, which can impact their decision to initiate PrEP [99]. It remains unclear which effect age may specifically have on the social norms held. Fifth, while we used existing scales and question formulations as a basis, our questions were iteratively formed in cooperation with community representatives. On the one hand, this likely led to questions better suited to the research context. On the other hand, not using fully pre-tested psychometric scales may have led to random error, with our measurement more sensitive to noise. Additionally, our results may be about narrower or different constructs than intended. However, given the extreme research fatigue of our target population [74], we chose to shorten scales and measure some concepts with single questions to achieve an adequate sample size and include multiple norm types in our design.

## Conclusion

The persistent PrEP gap among MSM is a challenge to ending the global HIV epidemic. This article addresses an important gap in empirical knowledge by identifying the relative importance of different social norms in PrEP-uptake intentions among MSM. Results show that attitude and role belief at the individual level, injunctive norms at the interpersonal level, and descriptive norms and self-comparison to peers at the community level are associated with PrEP intention. Addressing beliefs about attitudes and behavior of peers to impact the psychosocial path to PrEP uptake intentions is a necessary communication strategy in the effort to end the HIV epidemic. Further research is required on how liable these norms are to change and how they can be incorporated into PrEP interventions as mechanisms of change.

## Supplementary Information

Supplementary Material 1.

## Data Availability

The datasets analysed during the current study are available from the corresponding author on reasonable request.

## Abbreviations

AVE: Average Variance Detected
BM: Behavioral Model
CFA: Confirmatory Factor Analysis
CI: Confidence Interval
CFI: Comparative Fit Index
EMIS: European Men who have Sex with Men Internet Survey
HIV: Human Immune deficiency Virus
MSM: Men who have Sex with Men
PrEP: Pre-Exposure Prophylaxis
RMSEA: Root Mean Squared Error of Approximation
SE: Standard Error
SEM: Structural Equation Modeling
SNA: Social Norms Approach
SRMR: Mean Square Residual
TLI: Tucker Lewis Index
