# Supplementary material for: Prevention norms across socio-ecological levels and their relationship to PrEP-intention among men who have sex with men: a structural equation model
Source: BMC Public Health. 2026 Jul 22;26:2995. doi: 10.1186/s12889-026-28207-1 (PMC13679267; doi:10.1186/s12889-026-28207-1)
Supplement: Supplementary file 1 [file 12889_2026_28207_MOESM1_ESM.docx]

Appendix I Covariates

| Regression on intention to use PrEP | | | |
| --- | --- | --- | --- |
| Covariate | Estimate | T-value | P-value |
| Age Dummy | 0.006 | 0.058 | 0.954 |
| Urban environment Dummy | -0.005 | -0.048 | 0.962 |
| Education Dummy | -0.146 | -1.108 | 0.268 |
| More likely to encounter HIV Dummy | 0.389 | 3.440 | 0.001 |
